# Role of Technological Innovation in Achieving Social and Environmental Sustainability: Mediating Roles of Organizational Innovation and Digital Entrepreneurship

**DOI:** 10.3389/fpubh.2022.850172

**Published:** 2022-03-29

**Authors:** Daiyou Xiao, Jinxia Su

**Affiliations:** ^1^School of Finance, Central University of Finance and Economics, Beijing, China; ^2^School of Business, Central University of Finance and Economics, Beijing, China

**Keywords:** economic sustainability, social sustainability, digital entrepreneurship, organizational innovation, attitudes, technological innovation

## Abstract

Innovation has been a major growing driver of sustainability. The topic addressed in this study is a much-required transition to environmental and social sustainability considering the role of innovation in pacing up those changes. Digital evolution has greatly helped in dealing with climatic changes and promoting sustainability. This has helped the entrepreneurial organizations to adopt innovative approaches to tackle the inflexible challenges. Few developed and developing countries are at the forefront regarding technological innovation that encounter significant challenges in terms of innovation and adoption of new technologies and there is still a study vacuum as to whether the influence of technical innovation on achieving social and environmental sustainability differs depending on the stage of sustainability. This quantitative study has explored these effects collecting data from the SME's (small and medium enterprises). The findings of the study show that attitude toward technological innovation has a strong role to play in organizational innovation, digital entrepreneurship, environmental and social sustainability. Organizational innovation has been found a strong mediator between technological innovation and sustainability while digital entrepreneurship could not find significant results as mediator. This study will be useful for the countries and organizations involved in adopting new technologies considering their organization's role in achieving an overall eco-friendly and social sustainability.

## Introduction

Economic development has long been a focus of economic strategies, but it is only in the last few decades that sustainability has risen to the center of economic discourse and acquired substantial significance. Environmental conservation and social inclusion have made inroads into modern economics in recent times. Economic development cannot be sustained without social equality and environmental sustainability, it has become more obvious. Environmental sustainability is the route to the world we desire for everybody, stated former UN Secretary-General Ban Ki-moon. It provides a framework for achieving economic development, social fairness, environmental responsibility, and improved governance (1, 2). Economic, social, and environmental sustainability all fall under the umbrella of sustainable development (3, 4). Due to resource restrictions, conflicts between social, economic, and/or sustainable environmental objectives sometimes in between develops. However, according to modified neoclassical growth theories, in order to provide economic stability for future generations, it is critical to accomplish sustainability. Current policies, increased employment, and technological dissemination are all critical for sustainability performance.

Over time, environmental sustainability has become increasingly important. It instills a sense of resource scarcity and the importance of limiting environmental damage. It refers to decisions that will have an impact on all living things, environmental assets, and the environment (5). As a consequence, resource allocation must prioritize environmental sustainability. Biophysical boundaries, time dimension, social and moral systems, as well as uncertainties regarding technological advances and human wellbeing, must all be considered in future. Because some parts of harm to the natural ecosystem are permanent, achieving this goal is critical. Recent research suggests that environmental conservation, as well as human resources may be necessary preconditions for long-term economic success (6). Government, wealth inequality, property ownership, social inclusion, and empowering women are just a few of the elements that have a significant impact on a country's environmental sustainability.

It is often assumed that when people's fundamental requirements are met, they develop a desire to preserve the ecosystem. However, natural resources may have already been harmed at that point; consequently, it is critical that emerging countries recognize the need of environmental conservation (7). Environmental deterioration affects the whole planet, providing a health threat to many countries. As a result, it is critical to give it equal weight. With escalating levels of dynamic disaster risk coming from social polarization, rapidly rising poverty levels, urban conflict and violence, extremism, natural disasters, and climate change. Today's primary concerns are framed within the social context. These issues have an impact on planning and practices, prompting a reconsideration and reworking of present planning methodologies in order to address this terrible societal situation (8, 9). To establish strategies of sustainable development, it is widely agreed that a triad model, in which the ecological is intertwined with the economic and social, is essential. This three-pillar approach of sustainability has progressed significantly in terms of developing each part separately. According to the researchers, no definitive explanation of the link between the triad's pieces, or how they should be examined and evaluated, has been developed (10, 11).

Despite the fact that gaining this knowledge has profoundly impacted the sustainable discourse, one part of it, namely social sustainability, still lacks a cohesive, unambiguous, and usable definition (12). The flaws are usually attributed to social scientists, who are chastised for being conceptually ambiguous and inconsistent, resulting in a plethora of notions (13, 14). Furthermore, researchers discovered that the selection of social sustainability indicators is typically based on a practical grasp of plausibility and current political goals rather than theory. Every day, new technologies and ideas are developed and implemented across the world. Various organizations such as enterprises, universities, and research institutions, perform research and projects on a constant and relentless basis. As a result, a wide range of innovations are created, each with a different amount of originality, inventiveness, and knowledge. Any activity might result in a new discovery that advances scientific knowledge or the development of new technologies and breakthroughs.

Economic growth and its relevance in both emerging and developed countries may be explained by these accomplishments. Introducing new technology, without a question, elicits some opposition from the general people. Organizations and businesses alike find it difficult to accept dramatic changes that may affect their everyday lives or productivity (15–17). Adoption of new and unexpected technology advancements tends to produce self-consciousness inside organizations or businesses, resulting in a variety of attitudes (18, 19). Recognizing these distinctions can shed light on the important variables in technological innovation adaptation, as well as aid distinguish those factors for effective adoption procedures. Furthermore, these attitudes will result in varied degrees of technology acceptability in enterprises. Technological innovation is often related with science, method, or knowledge, and it focuses on the design and execution of something greater than what currently exists (20, 21). In recent years, technological innovation has become an essential component of scientific study due to its potential influence on the economics, community, and climate (22, 23). Improved organizational productivity will eventually result in more effective and efficient production, meeting the requirements of well-developed enterprises (24).

Organizational involvement with technology innovation has improved in the present knowledge-based sector (25). Owing to marketing, globalization, and shorter product cycles, competition in the industry is expanding. Several studies have looked at the obstacles and possibilities that come with adopting innovative technologies aimed at businesses and organizations, both of which have been examined at three levels: community, society, and lifestyles satisfaction (26). The findings provide incentive for businesses to create new management technology and processes. Despite first from apparent value of technical innovation, several studies show that it can also lead to uncontrolled growth. These elements have a significant impact on the ecosystem, causing environmental and socioeconomic issues. Enhanced resource consumption has a negative impact on the environment, whereas a shortage of such supplies has a negative impact on organizational performance, particularly in processes (26–28).

Natural resource depletion, pollutants, and environmental deterioration have changed the present flow of technological innovation and prompted the development of sustainable alternatives (3, 6). Organizations are urged to design, manufacture, and produce products and services that are both valued and sustainable in order to counteract environmental degradation; clients and consumers want such options in today's competitive global market (29). Technological innovation and organizational innovation plays a role in defining international performance of SMEs. The study focused on independent relationship of both of SMEs performance (30).

Despite the widespread interest in the role of technology innovation in supporting a sustainable society, the extant literature contains several theoretical and methodological limitations. Theoretically, scholars have focused on the weaker question, namely, whether technological innovation is compatible with the pillars of sustainability or not? Instead of looking at whether technological innovation is compatible with the pillars of sustainability, they have only considered the impact of technological innovation on each dimension apart from sustainability, rather than combining the two. Consequently, there is still a significant gap in technical innovation's potential to accomplish social and environmental sustainability in an integrated framework. Because a small number of developed and developing countries are at the forefront of technological innovation, many developing countries, particularly the least developed, geographically isolated, and tiny island developing states, tend to encounter significant challenges in terms of innovation and adoption of new technologies.

The independent variables of organizational innovation and technological innovation showed significant results toward SMEs performance (30), but left an open ended gap for exploration of the mediating relationship of these variables in achieving social and environmental sustainability regarding SMEs. This study based on the gap analyzed the mediating role of organizational innovation between technological innovation and socio-environmental sustainability. Digital entrepreneurship provides a platform for innovation to many entrepreneurial activities, it was identified that there could be a significant role of digital entrepreneurship in mediating the technological innovation for achieving sustainability (31). Moreover, no such research was found in past to determine the role of technological innovation in SMEs of China for achieving the social and environmental sustainability. To address these gaps and limitations due to empirical studies of specific regions, this research was designed with the following objectives.

1. To determine the role of attitude toward technological innovation on achieving social and environmental sustainability.
2. To evaluate the effects of mediation role between organizational innovation and digital entrepreneurship.

## Review of Literature and Hypotheses Development

**Figure 1:**
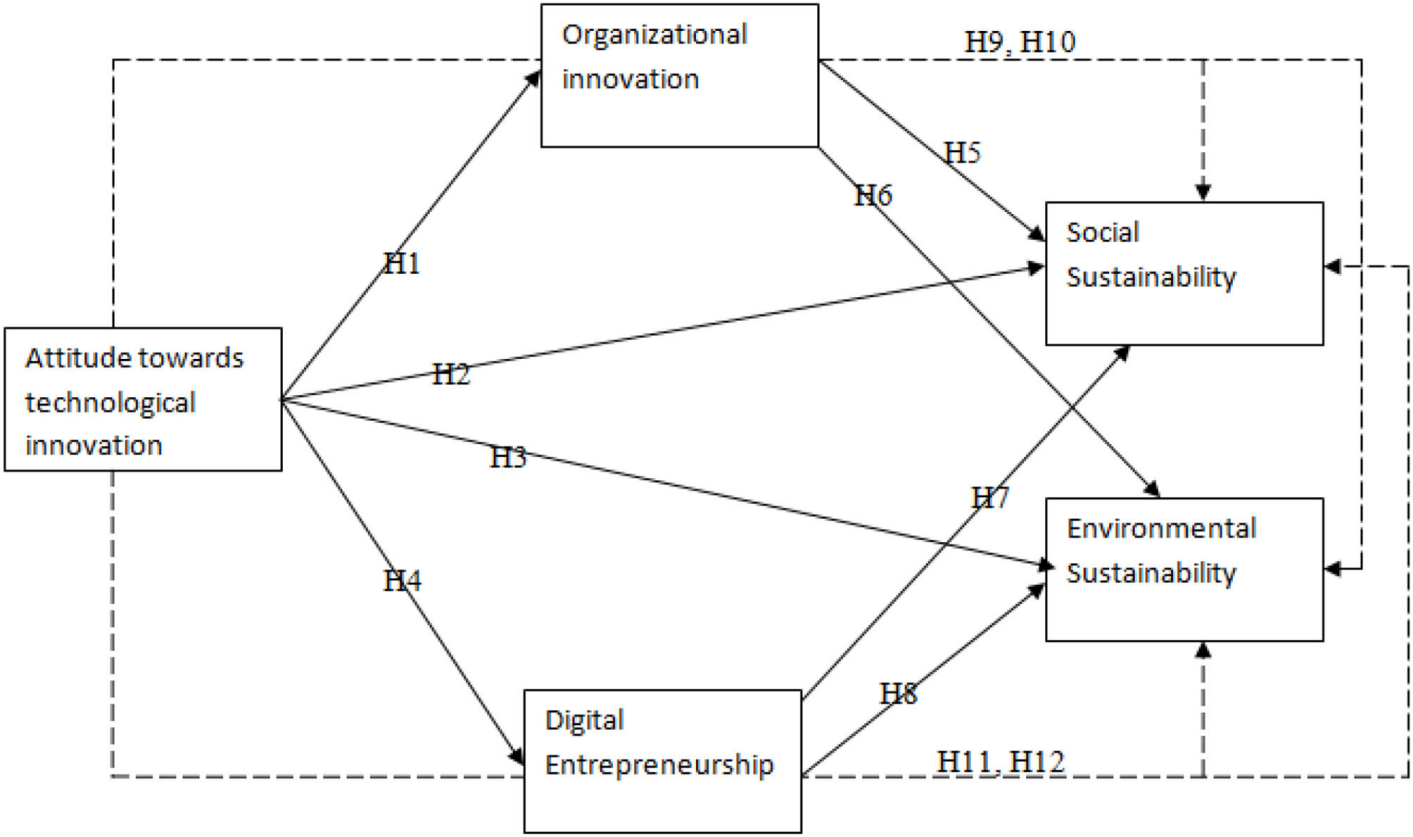
Conceptual model.

This research was based on identifying different technological innovation for achieving social and environmental sustainability. The effects of attitude toward technological innovation to achieve social and environmental sustainability were analyzed. Moreover, mediating roles of organizational innovation and digital entrepreneurship were also evaluated in this model (see Figure 1). This model is based on some theories of sustainability elaborated below.

### Diffusion of Innovation Theory

One of the earliest social science ideas is E.M. Rogers' Diffusion of Innovation (DOI) Theory, which he created in 1962. It was first used in marketing to describe how such an industry develops traction and disperses (or travels) through a population or social system over time. The eventual effect of this dissemination is that individuals embrace a new concept, habit, or product as part of a social structure. Adoption entails someone doing something unique from what they formerly did (purchase or use the new product, acquire and perform a new behavior, etc.). Adoption depends on the perception of the concept, behavior, or commodity as novel or unique. Diffusion is conceivable as a result of this (22). Diffusion, according to Rogers, is the process through which an invention is disseminated among the members in a social system over time. The spread of innovations hypotheses has a wide range of sources that span different fields (32). Numerous researchers have incorporated broad diffusion theories of technological innovation based on Rogers' theory to adopt and diffuse an innovation technology in higher education institutions at both the macro and micro levels.

Rogers' theory has been frequently employed in this respect to explain why the acquisition and spread of innovation differs among societies. Diffusion, as per Rogers, is “the process by which an invention gets disseminated through particular channels among the members of a social system over time,” where the “innovation” might be anything that the adopters perceive as novel. It is the most often cited theory in the subject of innovation dissemination, and it is comprised of four key factors of spread: invention, channels of communication, timing, and the social structure (23). Many investigations, hypotheses, and models in the subject of diffusion have been based on this principle. Rogers' idea has been utilized in a number of researches to explain why some people absorb technology innovation while others do not. In this regard, Rogers claims that everyone follows a normal distribution when it comes to technology adoption and diffusion. This suggests that there is a bias toward the necessity to disseminate technology without taking into account the repercussions of doing so (33).

Furthermore, when an invention fails, the early adopters are sometimes blamed instead of other aspects in the diffusion process, such as the social structure in which they operate or the qualities of the technology they utilize. Rogers claims that it is an example of individual blame bias since technical progress has a “propensity to agree with agents of change who advocate innovations rather than the audience of prospective adopters.” Personal adopters as participants in a social system, according to Rogers, need not to accept an invention in the same way. Change initiatives are focused on quick adoption and dissemination in order to get instant outcomes without contemplating the ramifications on the social structure. Rogers' social system has three basic keywords or concepts: adaptability with technology, and tech enthusiasts (34). So, this theory provided basis for technological innovation (35, 36), which leads to organizational innovation and aid in achieving sustainability (37, 38), social or environmental.

### Relationship of Attitude Toward Technological Innovation With Socio-Environmental Sustainability and Mediators

Attitudes may be developed internally and/or persuaded with enough effort and time (39). Attitudes as a judgment process influenced by emotional, opinions, and actions, which they referred to as internal attitudes (40). Theme of the story is sometimes referred to as an internal attitude since it involves personal gains for personal ideas (41). Externally, persuasion based on credibility, sympathy, and logic will refine and impose arguments on people's attitudes, affecting their moral duty (42). In addition to the antecedents, external influences such as eco-centrism and altruism are thought to impact attitudes (43). Those working on sustainability studies have a diverse range of views on technology. People range from those who see innovation efficiency gains as the feature to bring up sustainability issues to those who see them as the root of the problem; increasing absolute resource consumption through rebound effects and speeding up the disruption of natural ecosystem cycles by introducing ever more alien substances. There are countless and at first glance perplexing permutations and combinations of attitudes in between these two situations (44).

Translating information into economic activity is what innovation is all about. It is a multi-source activity of discovering, understanding, and using new technologies and processes. This is a key driving force of economic and performance improvement and, as a result, of higher living standards (45). Since the 1820's, when Joseph Schumpeter first proposed the notion of innovation, the performance of technology innovation, the separation of innovation stages, the characteristics of technology innovation, as well as its role have received a lot of attention. The link among innovation and location has become a major focus of economic growth research in the last two decades. SMEs will confront harsh competitiveness from local and international enterprises in their traditional home markets, as well as in their export industries, as a result of increased globalization and different rules of the WTO framework. In order to establish core competencies in industry, technology is essential. Technology innovation is seen as a means of boosting an economic growth of the country. SMEs are the driving factor behind technological advancement (45).

Organizational innovation explains the application of new and/or improved ideas and processes within the company's workplace, including such marketing and management systems to cost savings and create value for the company and other external stakeholders, whereas technological innovation deals with the introduction of new products and processes directly for clients or customers (46, 47). The existing research usually treats technical and organizational innovation as two distinct factors that influence organizational effectiveness. It is less clear, however, how SMEs might profit from the synergy of these two innovative capacities during internationalization. Using a combination of technology and organizational advances to learn more about the dynamics of the foreign markets in which they operate provides a learning platform for businesses. Technological and organizational innovation work in tandem to help SMEs in emerging economies improve their international performance (30). This suggested a possible relationship between technological innovation and organizational innovation. Several other relationships in this regard between attitudes toward technological innovation, digital entrepreneurship and socio-environmental sustainability could have been developed as suggested by (48, 49). So, we developed the following hypotheses.

***H***_**1**_*: Attitude toward technological innovation has an effect on organizational innovation*.
***H***_**2**_*: Attitude toward technological innovation has an effect on social sustainability*.
***H***_**3**_*: Attitude toward technological innovation has an effect on environmental sustainability*.
***H***_**4**_*: Attitude toward technological innovation has an effect on digital entrepreneurship*.

### Relationship Between Organizational Innovation and Socio Environmental Sustainability

Organizational innovation is sometimes referred to as administrative or management innovation, and it may be defined as “how managers do what they do.” Researchers defined administrative innovation as “new ideas for the recruitment of people, the distribution of resources, and the structure of duties, authority, and incentives,” as opposed to “technological innovation (50, 51).” Changes in organizational structures, changes in people's behaviors and attitudes, and new rules, roles, and processes are all examples of organizational innovation (52). Some people think of organizational innovation as a result of product innovation, while others think of it as a process. Researchers have associated the foregoing viewpoints by suggesting that innovation should be characterized in terms of both goods and processes, as well as the integration of processes and outcomes (53, 54).

Others argue that in the past, the emphasis was only on “technical innovation” in goods, procedures, and technology. Some argue that management innovation should be included in the notion of organizational innovation. The notion of organizational innovation has become more wider in recent years (55). Yet, much of the available research divides it into only two categories: management and technological innovation (which includes processes, strategies, organizations, strategies, and operations). A few studies have been conducted in past to analyze the relationship of organizational innovation with environmental sustainability and found significant relationship between the both such as (56). This suggested a possible relationship in different perspectives as well. The mediating role of organizational innovation has also been studied in many contexts such as (57), in which partial mediation was found between strategic agility and firm performance.

Another study analyzed the mediating impact of organizational innovation between change oriented leadership and the performance of the organizations by (58). The study concluded that university performance was directly facilitated by the organizational innovation. Another evidence was found for its mediating role toward organizational performance with the entrepreneurial leadership by (59). The results were significant and proved its role as a mediator between these aspects. Although there is not much evidence on mediating role of organizational innovation between technological innovation and the social and environmental sustainability, but these previous findings suggested a probable role of being mediator between such relationships. Hence, we formulated the following hypotheses in achieving social and environmental sustainability in SMEs of China.

***H***_**5**_***:*** *Organizational innovation has an effect on social sustainability*.
***H***_**6**_***:*** *Organizational innovation has an effect on environmental sustainability*.
***H***_**9**_***:*** *Organizational innovation mediates the relationship of attitude toward technological innovation and social sustainability*.
***H***_**10**_***:*** *Organizational innovation mediates the relationship of attitude toward technological innovation and environmental sustainability*.

### Relationship Between Digital Entrepreneurship and Socio Environmental Sustainability

Governments, companies, and organizations are adopting or increasing their usage of digital technology such as cloud technology, machine learning, 3d printers, and edge technology (60). Digital opportunities relate to new opportunities for action in respect to a given user or usage environment that may be used by entities such as entrepreneurs due to the distinctive qualities of digital technology (61, 62). The journey of digitalization results in innovative institutional mechanisms, introducing unique values, practices, and institutions that change the game's existing norms and challenge current logic configurations (63). Generally recognized and configurable digital components, like as ERP (enterprise resource planning) systems, or standard-setting digital infrastructures that coordinate the interaction of players, such as product portals and blockchain technology, are examples of these setups. Importantly, these powerful digital developments have an impact on business structures. Scholars contend that the technological affordances that come with digital infrastructures and modules expand the alternatives for generating, delivering, and collecting value and create new paths for doing so (63, 64). Economic transformation results in radically new company models that require certain organizational capacities to be achieved successfully (65). Digital technologies potentially use their own but nevertheless growing logic which exists alongside with and affects the perception and execution of previous interpretations by bringing new behaviors, attitudes, and systems (66).

Digitization develops all around notions of connectedness, accessibility, availability, access, adaptability, and inheritability (62, 67). As digital entrepreneurship is relatively newer dimension in achieving sustainability of the organizations socially and environmentally, it has great scope for tackling the climate change and social setups problems. Innovative responses to apparently intractable societal concerns have been implemented by digital technology in entrepreneurial enterprises as suggested by (68). These issues pertain to digital sustainability in general. It presents a research agenda that creates unique concerns for entrepreneurship, marketing strategies, and environments, as well as new ways of thinking about trust and institutional logics, by concentrating on the digital toolset used by pioneering businesses. Digital entrepreneurship has also been studies as a mediator between organizational structures in recent past (69). It suggested its role of being mediator in our concept of achieving socio-environmental sustainability by utilizing the technological innovations. More of the research has generally focused on mediating roles of digital technologies for digital entrepreneurship instead of evaluating digital entrepreneurship as mediators. Based on the above discussion and probable relationships of digital entrepreneurship, we suggest the following hypotheses.

***H***_**7**_***:*** *Digital entrepreneurship has an effect on social sustainability*.
***H***_**8**_***:*** *Digital entrepreneurship has an effect on environmental sustainability*.
***H***_**11**_***:*** *Digital entrepreneurship mediates the relationship of attitude toward technological innovation and social sustainability*.
***H***_**12**_*: Digital entrepreneurship mediates the relationship of attitude toward technological innovation and environmental sustainability*.

## Methodology

In this quantitative study, the owners of small and medium enterprises (SMEs) situated in China had participated in this study were approached in the duration of 2 months of November and December 2021. It was a deductive approach following a post-positivism philosophy of research measuring the effect of independent variables on dependent variable. A convenience sampling was used as the respondents were approached as per the researcher's feasibility considering a large number of SMEs, also this is most commonly used sampling technique (70). The total number of usable questionnaires returned from 400 distributed questionnaires was 314 making a response rate of 78.5%. The received questionnaires were filtered for the missing data or outliers. All the items representing variables of the study were measured on a five-point Likert scale.

### Instrument Development

The instrument used in the present study for data collection was questionnaire survey. Questionnaire used in this study was consist of 36 items in total which were divided into six sections. The first section of the questionnaire indicated the options for the demography of the respondents and rest of the five sections addressed the independent, mediating and dependent variables of the study. It included age, gender, education and the age of their organization. The second section of the questionnaire included the scale of attitude toward technological innovation which had 10 items adapted from (71). The second section had the mediating variable of organizational innovation consisting of 6-item taken from (72). The second mediating variable of digital entrepreneurship was adapted from (73) which consisted of 10 items. The dependent variables of environmental sustainability mentioned in the fifth and sixth sections, respectively had five items each that were adapted from (74).

### Demographics Details

First, the data of demography of the respondents was analyzed with frequency and percentage. The responses for gender were categorized into males and females. Out of the total respondents, 165 were male and 149 were females. As for age, the highest number of respondents were age of 31 years and above followed by the category 26–30 year age (92 respondents) and category 21–25 year age (64 respondents). The responses showed that 32 respondents had Ph.D degree or other diplomas while highest number of respondents had master's degree (230 respondents) followed by bachelors degree (52 respondents). The highest number of SME's had been operation for <5 years (132 respondents), 96 owners of SME's had been working for 11 or more years. The details are given in the Table 1.

**Table 1:** Demographics analysis.

**Demographics**	**Frequency**	**Percentage**
**Gender**		
Male	165	52.54%
Female	149	47.45%
**Age**		
15–20	15	4.77%
21–25	64	20.38%
26–30	92	29.29%
31 and above	143	45.54%
**Education**		
Bachelors	52	17.19%
Masters	230	73.24%
Ph.D. and others	32	10.19%
**Age of organization (years)**		
1–5	132	42.03%
6–10	86	27.38%
>11	96	30.57%

## Data Analysis and Results

Smart-PLS version 3 has been used for the analysis of the data based on the partial least square structural equation modeling (PLS-SEM). In this software, the data analysis is done through measurement model estimation and structural model estimation. In measurement model, reliability and validity of the data obtained are checked using the tests Heterotrait monotrait (HTMT) ratio, Fornell and Larcker criteria, average variance extracted (AVE), Cronbach alpha and composite reliabilities. In structural model, the data is run for the rejection or acceptance of the hypotheses. The statistics used in this regard are t-statistics, *p*-values.

### Measurement Model

**Figure 2:**
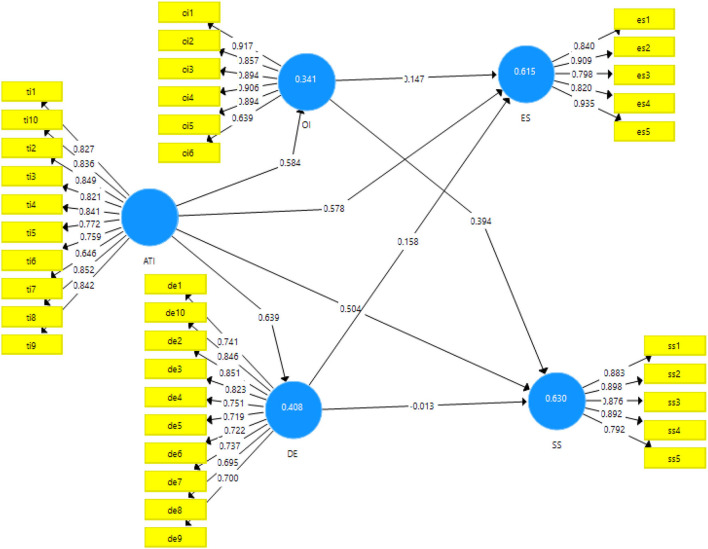
Output of measurement model. ATI, Attitude toward technological innovation; OI, Organizational innovation; DE, Digital entrepreneurship; ES, Environmental sustainability; SS, Social sustainability.

The algorithm obtained from the measurement mode is presented in Figure 2.

Results of factor loadings, AVE, Composite and Cronbach alpha reliabilities have been mentioned in the Table 2. All the values of the factor loadings are above 0.60 which is the threshold and reliabilities are above 0.7 that is the cut off value (75). The minimum value of the study obtained for reliabilities is 0.918. AVE is ideally supposed to be more than 0.5, and all the values in this study were reported above the threshold.

**Table 2:** Factor loadings, AVE and reliabilities.

**Variables**	**Factor**	**Loadings**	**Cronbach alpha**	**Composite reliability**	**AVE**
Attitude toward	ti1	0.827	0.940	0.949	0.651
technological innovation	ti10	0.836			
	ti2	0.849			
	ti3	0.821			
	ti4	0.841			
	ti5	0.772			
	ti6	0.759			
	ti7	0.646			
	ti8	0.852			
	ti9	0.842			
Digital entrepreneurship	de1	0.741	0.920	0.932	0.579
	de10	0.846			
	de2	0.851			
	de3	0.823			
	de4	0.751			
	de5	0.719			
	de6	0.722			
	de7	0.737			
	de8	0.695			
	de9	0.700			
Environmental sustainability	es1	0.837	0.913	0.935	0.743
	es2	0.910			
	es3	0.798			
	es4	0.822			
	es5	0.935			
Organizational innovation	oi1	0.917	0.924	0.942	0.734
	oi2	0.857			
	oi3	0.894			
	oi4	0.906			
	oi5	0.894			
	oi6	0.639			
Social sustainability	ss1	0.883	0.918	0.939	0.755
	ss2	0.898			
	ss3	0.874			
	ss4	0.893			
	ss5	0.793			

The discriminant validity of the data for the present study has been checked with HTMT ratio and Formell and Larcker criteria. The Table 3 shows the results for HTMT ratio. The acceptance criteria for HTMT ratio is that all the values present in the grid should be <0.85 (76). The highest value obtained for this grid is 0.817 which is below the threshold. Similarly, the other test used is the Fornell and Larcker criteria. The acceptance criteria for Fornell and Larcker criteria are that the top most value in each column should be the highest. The available table for results shows that this criteria is met in Table 4.

**Table 3:** Fronell and Larcker criteria.

	**ATI**	**DE**	**ES**	**OI**	**SS**
ATI	0.807				
DE	0.639	0.761			
ES	0.763	0.588	0.862		
OI	0.584	0.432	0.551	0.857	
SS	0.727	0.479	0.602	0.684	0.869

*ATI, Attitude toward technological innovation; OI, Organizational innovation; DE, Digital entrepreneurship; ES, Environmental sustainability; SS, Social sustainability*.

**Table 4:** HTMT ratio.

	**ATI**	**DE**	**ES**	**OI**	**SS**
ATI					
DE	0.650				
ES	0.817	0.601			
OI	0.626	0.444	0.591		
SS	0.776	0.488	0.649	0.743	

*ATI, Attitude toward technological innovation; OI, Organizational innovation; DE, Digital entrepreneurship; ES, Environmental sustainability; SS, Social sustainability*.

Additionally, the r-square values for the social sustainability variable have been found the highest 63% followed by environmental sustainability 61%. Digital entrepreneurship has explained the model fit 40% while organizational innovation has shown the r-square values of 34%.

### Structural Model

**Figure 3:**
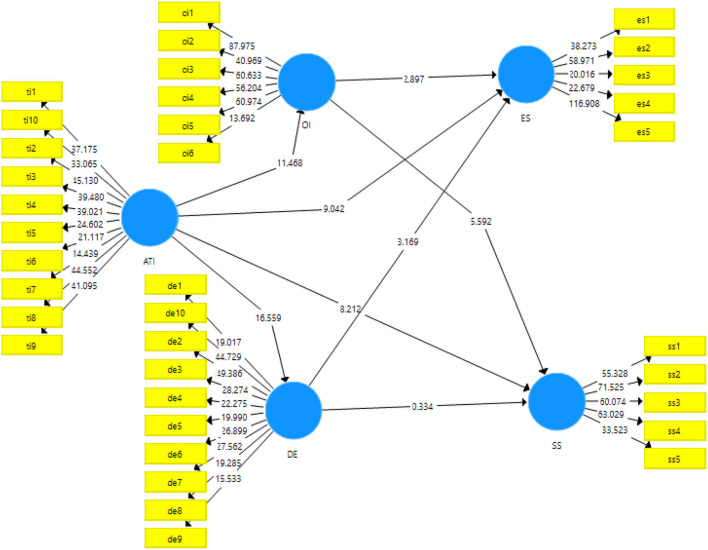
Output of structural model. ATI, Attitude toward technological innovation; OI, Organizational innovation; DE, Digital entrepreneurship; ES, Environmental sustainability; SS, Social sustainability.

The hypotheses of the study are accepted or rejected based on the results obtained from structural model in Smart-PLS. The acceptance criteria of the hypotheses are based on the t-statistics and *p*-values. The output for the structural model estimation has been given in the Figure 3.

The results for the direct relationships of the study have been given in the Table 5. All the hypotheses of the study have been set for acceptance at *p* < 0.05. There are total eight direct relationships of the study; of which seven have been accepted and H_7_ regarding the effect of digital entrepreneurship on social sustainability has been rejected while rest of the variables have shown significant effects and t-statistics.

**Table 5:** The direct effects of the variable.

**Paths**	**H**	**O**	**M**	**SD**	**T-Statistic**	***P*-value**	**Results**
ATI → OI	H_1_	0.584	0.588	0.049	11.860	0.000***	Accepted
ATI → SS	H_2_	0.504	0.506	0.063	8.029	0.000***	Accepted
ATI → ES	H_3_	0.578	0.574	0.063	9.109	0.000***	Accepted
ATI → DE	H_4_	0.639	0.642	0.039	16.448	0.000***	Accepted
OI → SS	H_5_	0.394	0.390	0.070	5.665	0.000***	Accepted
OI → ES	H_6_	0.147	0.152	0.053	2.758	0.006*	Accepted
DE → SS	H_7_	−0.013	−0.008	0.039	0.321	0.749	Rejected
DE → ES	H_8_	0.158	0.156	0.047	3.325	0.001**	Accepted

p*** < 0.001, p** < 0.005, p* < 0.05, H, Hypotheses; O, Original Sample; M, Sample Mean; SD, Standard Deviation; ATI, Attitude toward technological innovation; OI, Organizational innovation; DE, Digital entrepreneurship; ES, Environmental sustainability; SS, Social sustainability.

The first hypotheses (H_1_) regarding the effect of attitude toward technological innovation has been accepted (β = 0.58, t-statistics = 11.86, *p* < 0.001). The second hypotheses (H_2_: attitude toward technological innovation has an effect on social sustainability; β = 0. 50, t-statistics = 8.029, *p* < 0.001) and third hypotheses (H_3_: attitude toward technological innovation has an effect on environmental sustainability; β = 0.57, t-statistics = 9.109, *p* < 0.001) also showed significant results. The fourth hypotheses (H_4_) regarding the direct effect of attitude toward technological innovation has also been accepted (β = 0.63, t-statistics = 16.44, *p* < 0.001). The direct effect of organizational innovation has also been found significant on social sustainability (H_5_: organizational innovation has an effect on social sustainability; β = 0.39, t-statistics = 5.66, *p* < 0.001) and environmental sustainability (H_6_: organizational innovation has an effect on environmental sustainability; β = 0.14, t-statistics = 2.75, *p* < 0.05) have also been approved. Similarly, digital entrepreneurship could find significant effect on environmental sustainability (β = 0.39, t-statistics = 5.66, *p* < 0.005).

There are total four indirect or mediating effects measured in this study. The two hypotheses for the mediating role of organizational innovation have been approved i.e., H_9_: organizational innovation mediates the relationship of attitude toward technological innovation and environmental sustainability (β = 0.08, t-statistics = 2.42, *p* < 0.05) and H_10_: organizational innovation mediates the relationship of attitude toward technological innovation and social sustainability (β = 0.23, t-statistics = 5.66, *p* < 0.001) have been approved with significant results. The first mediation of digital entrepreneurship regarding environmental sustainability has been approved (β = 0.10, t-statistics = 3.13, *p* <0.05) while the other mediation has been rejected based on the results obtained. The indirect effects of the variables have been reported in Table 6.

**Table 6:** The indirect effects of the variable.

**Paths**	**H**	**O**	**M**	**SD**	**T-Statistic**	***P*-value**	**Results**
ATI -> OI -> ES	H_9_	0.086	0.090	0.035	2.423	0.016*	Accepted
ATI -> OI -> SS	H_10_	0.230	0.228	0.041	5.633	0.000**	Accepted
ATI -> DE -> ES	H_11_	0.101	0.100	0.032	3.134	0.002**	Accepted
ATI -> DE -> SS	H_12_	−0.008	−0.005	0.025	0.320	0.749	Rejected

p** < 0.005, p* < 0.05, H, Hypotheses; O, Original Sample; M, Sample Mean; SD, Standard Deviation; ATI, Attitude toward technological innovation; OI, Organizational innovation; DE, Digital entrepreneurship; ES, Environmental sustainability; SS, Social sustainability.

## Discussion

This innovative research in the field of technological innovation provides as excellent platform for digital entrepreneurs of SMEs of China and also provides useful insights about organizational activities to the digital entrepreneurs worldwide to achieve social and environmental sustainability goals. This research was divided in to two segments of evaluation. In first part direct relationships of organizational processes were analyzed and in the other part indirect or mediated relationships were analyzed. The results showed some interesting facts and proved the vitality of this research model. Attitudes toward technology and innovative technology play an important role in adoption for technologies for innovative businesses. In this case of SMEs in China, they also had a lot to offer as technology innovation is seen as a means of boosting an economic growth of the country. SMEs are the driving factor behind technological advancement (45). The first hypotheses was about effects of attitude toward technological innovation with organizational innovation which was significant.

A lot of researchers in the past have studied the both as one unit considering technological innovation an integral part of organizational innovation but many of them oppose the connection between them and consider them totally apart from each other (51, 55, 56). Not even a single research analyzed the impact or effects of one on another. The results of our study would provide a connecting link between the both for future researchers. The second hypotheses was about effects of attitude toward technological innovation on social sustainability. The results showed significant effects of the former on the later one. This indicated that behavioral modification for attaining a specific attitude toward technological innovation could produce positive impact on doing the entrepreneurship leading to achieving social sustainability which is considered as management of social setup conducive for performing the entrepreneurship. The previous researches didn't identify the direct role of attitude toward technological innovation with social sustainability. The third and fourth hypotheses were about the effects of attitude toward technological innovation and digital entrepreneurship and environmental sustainability.

These hypotheses were also accepted and produced significant results. This indicated a direct relationship of attitudes toward technological innovation with all these four variables. The possible reasoning is confined to the development of attitudes for certain things which is a behavioral process and a lot of researchers of the past have focused on this aspect as well where attitudes play important roles in developing a sense of innovation. These relationships were not analyzed before but several other relationships in this regard between attitudes toward technological innovations, digital entrepreneurship and socio-environmental sustainability were developed in this study as suggested by (48, 49). This research would yield positive contribution while devising the relationship of these processes.

The fifth and sixth hypotheses were about the direct relationships of organizational innovation with social sustainability and environmental sustainability. These hypotheses yielded significant results and proved a direct and strong relationship to achieve the sustainability. These kind of relationships in different perspectives were also studied before in which impact of organizational innovation was studied on achieving sustainable development as both these kinds of sustainability are the components of sustainable development (60, 61). This could be the nature of organizational innovation as organizational innovation provide sustainable measures for achieving sustainability in the organizations like SMEs. The seventh and eighth hypotheses were about the direct relationship of digital entrepreneurship with social sustainability and the environmental sustainability. The results of seventh hypotheses were non-significant and significant for the eight hypotheses, indicating a strong relationship with environmental sustainability and no relationship with social sustainability.

As digital entrepreneurship is relatively newer dimension in achieving sustainability of the organizations socially and environmentally, it has great scope for tackling the climate change and social setups problems. Innovative responses to apparently intractable societal concerns have been implemented by digital technology in entrepreneurial enterprises as suggested by (68). Digital entrepreneurship was not directly studied for both components of sustainability before but our study indicates that impact of digital entrepreneurship would be bifacial in making environment more sustainable while performing organizational processes in SMEs of China. The indirect relationship of digital entrepreneurship with other processes were suggested by (69). Based on these suggestion, mediating role of digital entrepreneurship was studied in this research.

The mediating roles of digital entrepreneurship yielded mixed results as digital entrepreneurship did not have direct relation with social sustainability mentioned previously, it also did not have any mediation between attitude toward technological innovation and social sustainability. Although, direct relationship between the both was significant indicating no need of mediation for achieving social sustainability but the mediating link of digital entrepreneurship suggested by (69), was significant between attitude toward technological innovation and environmental sustainability. The direct relationship was also significant and proved the authenticity of digital entrepreneurship in achieving environmental friendly sustainability of the organizations as, digital entrepreneurship contribute very less pollution to the environment in comparison to existing organizational setups not utilizing digital means on mass level.

The mediating roles of organizational innovation were also studied in this research which provided full mediation between attitude toward technological innovation and the social and environmental sustainability. The mediating role of organizational innovation were also studied before in past by (62–64). The outcomes of these researchers indicated that organizational innovations for achieving sustainability in the organizations was significant for providing an indirect approach for achieving the goals. Our research also found significant results in terms of organizational innovation as it would enhance the impact of attitude toward technological innovation toward achieving social and environmental sustainability of SMEs.

## Theoretical Implications

Many research studies have been conducted in the past that have considered the technological and organizational innovation as overlapping concepts, however, it had been opposed by few researchers as suggested by (50, 51). No research in the past had analyzed the impact of these two variables on one another. The results in this study provide a link between these two variables. Secondly, the effect of attitude toward technological innovation on digital entrepreneurship and environmental sustainability confine the development of attitudes for certain things which are the behavioral aspect, and a lot of researchers have focused on this aspect as well, where attitudes play important roles in developing a sense of innovation. However, these specific relationships were not analyzed before. Hence, in this regard this study has attempted to measure the relationships between attitudes toward technological innovations, digital entrepreneurship, and socio-environmental sustainability.

## Limitations and Future Directions

Despite novelty of the study, there have been few limitations in the study. First of all, the owners of the SME's have been taken as population samples; however, this study should be generalized considering the view of employees working in organizations to get neutral and unbiased responses regarding these variables. Secondly, measurement of attitude toward organizational innovation is a new concept as these had been considered overlapping the past researchers, therefore, further validation is required in future if these are mutually exclusive processes as suggested by present study. Third, the major component of the sustainability is missing in this study, future studies should focus on the proposition if economic sustainability is also affected by the predicting variables used in this study. Furthermore, the moderating roles of organizational climate, brand equity, job satisfaction and self-efficacy are missing that can be checked in the future studies.

## Conclusion

A number of co-existing phenomenological perspectives have a distinctive characterization on how to cope with the sustainable development, ecological and climatic changes and creating socio-ecological changes. In this study, the lens of attitude toward technological innovation has been added focusing on the entrepreneurial and organizational innovative perspectives to attain environmental and social sustainability. The study has shown interesting results from the data obtained from the owners of SMEs in China. The data obtained from the respondents had been screened for validity and reliability of data then the hypotheses were tested using Smart-PLS structural equation modeling (SEM). The results had been interesting showing attitude toward technological innovation has found strong positive effects on organizational innovation, digital entrepreneurship, social and environmental sustainability. Individually, organizational innovation had been a strong mediator and predictor of social and environmental sustainability while digital entrepreneurship could not find significant results.

## Data Availability Statement

The original contributions presented in the study are included in the article/supplementary material, further inquiries can be directed to the corresponding author.

## Ethics Statement

The studies involving human participants were reviewed and approved by Central University of Finance and Economics China. The patients/participants provided their written informed consent to participate in this study. The study was conducted in accordance with the Declaration of Helsinki.

## Author Contributions

DX conceived, designed the concept, and wrote the paper. JS helps in data collected and editing. Both authors read and agreed to the published version of the manuscript.

## Conflict of Interest

The authors declare that the research was conducted in the absence of any commercial or financial relationships that could be construed as a potential conflict of interest.

## Publisher's Note

All claims expressed in this article are solely those of the authors and do not necessarily represent those of their affiliated organizations, or those of the publisher, the editors and the reviewers. Any product that may be evaluated in this article, or claim that may be made by its manufacturer, is not guaranteed or endorsed by the publisher.
